# Is a White Diet Necessary for Tooth Bleaching Procedures? A Systematic Review and Meta-Analysis

**DOI:** 10.3390/dj12040118

**Published:** 2024-04-22

**Authors:** Louis Hardan, Rim Bourgi, Abigailt Flores-Ledesma, Walter Devoto, Emma Devoto, Miguel Ángel Fernández-Barrera, Naji Kharouf, Carlos Enrique Cuevas-Suárez

**Affiliations:** 1 Department of Restorative Dentistry, School of Dentistry, Saint-Joseph University, Beirut 1107 2180, Lebanon; louis.hardan@usj.edu.lb (L.H.); rim.bourgi@net.usj.edu.lb (R.B.); 2 Department of Biomaterials and Bioengineering, INSERM UMR_S 1121, Biomaterials and Bioengineering, 67000 Strasbourg, France; naji.kharouf@etu.unistra.fr; 3 Dental Materials and Biomaterials Laboratory Faculty of Stomatology, Meritorious Autonomous University of Puebla, Puebla 72000, Mexico; abigailt.flores@correo.buap.mx; 4 Private Practice, 16030 Liguria, Italy; walter@walterdevoto.com (W.D.); emma@walterdevoto.com (E.D.); 5 Dental Materials Laboratory, Academic Area of Dentistry, Autonomous University of Hidalgo State, Circuito Ex Hacienda La Concepción S/N, San Agustín Tlaxiaca 42160, Mexico; miguel_fernandez10334@uaeh.edu.mx; 6 Department of Endodontics and Conservative Dentistry, Faculty of Dental Medicine, University of Strasbourg, 67000 Strasbourg, France

**Keywords:** enamel color change, free-staining diet, at-home bleaching, in-office bleaching, color difference

## Abstract

The aim of this investigation was to conduct a systematic review and meta-analysis to determine the necessity of a white diet during or following a bleaching procedure. This systematic review and meta-analysis followed the PRISMA guidelines meticulously. The research question was: Is a white diet necessary during and/or after a bleaching treatment? In vitro studies or clinical trials reporting the color change in bleached enamel after the use of a free-staining diet were considered for full-text review. For the analyses, a random-effects model was employed. Statistical significance was defined as a *p*-value < 0.05. A total of 17 documents were eligible for qualitative analysis: 5 clinical trials and 12 in vitro studies. Only data from the clinical trials were included in the meta-analysis. For at-home bleaching, differences in the color among the subjects were not statistically significant during the first (*p* = 0.64), second (*p* = 0.26) or third (*p* = 0.43) weeks of treatment. Also, the color difference one month after finishing the bleaching treatment were not statistically significant (*p* = 0.27). The color difference one month after finishing an in-office treatment showed that the restrictions on diet did not significantly improve the bleaching outcomes (*p* = 0.90). According to the findings of this review, dietary restrictions are not necessary during or after bleaching procedures.

## 1. Introduction

Patients experiencing tooth discoloration often choose to undergo teeth whitening procedures [1]. Achieving the desired outcome in dental bleaching requires an accurate diagnosis of the specific type of staining present on the tooth surface [2]. The two main categories of tooth staining sources are intrinsic and extrinsic staining [3,4]. Extrinsic stains typically originate from external sources and are linked to pigmented dyes found in substances like tobacco, certain medications, and the buildup of bacterial plaque. While superficial stains can typically be effectively removed through prophylactic measures like professional cleaning [5], intrinsic stains result from changes in the structural composition or thickness of dental hard tissues [6,7]. This entails the integration of chromogenic material within dental hard tissue, occurring during tooth development [8]. To address these discolorations, tooth bleaching techniques are employed.

Dental bleaching encompasses a range of techniques and concentrations of bleaching gel, with in-office treatments, at-home solutions, or a combination of both being the most prevalent and effective methods tailored to individual patient needs and staining severity [1,3,9,10]. While at-home tooth whitening involves the daily application of lower concentrations of carbamide peroxide (CP), in-office whitening employs higher concentrations of bleaching gels. The choice between these approaches hinges on treatment goals, patient preferences, and clinical considerations. In-office procedures typically produce faster and more noticeable results, often incorporating techniques like light activation. Conversely, at-home kits offer the convenience of home-based treatment and may be more cost-effective. Both approaches are generally safe when used as directed, with in-office treatments benefiting from professional supervision and customization. Decisions regarding the use of light activation should be made in consultation with a dental professional, considering individual patient factors and preferences, while prioritizing safety and efficacy [1,2,5,7].

However, for safe light usage, it is crucial to use a device with lower power density and activation spacing. This allows an adequate cooling time for the tooth structure and reduces the risk of postoperative sensitivity and pulp issues [11,12,13]. However, due to shorter dentist schedules, lower patient costs, and lower risk of tooth sensitivity following the process, at-home tooth whitening is successful and widely encouraged [14].

Professional guidelines for teeth bleaching recommend that patients should cease smoking and avoid consuming foods high in coloring agents, such as wine, soft drinks, tea, and coffee, in order to adopt a “white diet” [14]. Prior research indicates that the loss of organic material on the enamel surface after teeth whitening results in a surface with porosities and irregularities, which can lead to increased extrinsic coloration [15]. Additionally, in these conditions, consuming foods high in coloring agents may further alter the structure of the bleached enamel [16,17], making it more susceptible to staining directly following the whitening process [18,19]. Moreover, certain acidic foods and beverages can erode the enamel, exposing the underlying dentin and increasing the risk of discoloration [20,21]. Consequently, specific ingredients from the diet might compromise the long-lasting outcome of the bleached tooth, resulting in stains both during and after the procedure [15]. Therefore, it is essential for patients to adhere to post-bleaching dietary recommendations to maintain the aesthetic results of the procedure.

In addition to dietary considerations, proper oral hygiene practices play a crucial role in maintaining the results of dental bleaching. Regular brushing with a fluoride toothpaste and flossing help to remove surface stains and prevent the buildup of plaque and tartar, which can contribute to discoloration over time. Furthermore, routine dental visits for professional cleanings and check-ups allow dentists to monitor the condition of the teeth and provide additional recommendations for maintaining a bright, healthy smile. By combining dietary modifications with diligent oral care, patients can optimize the effectiveness and longevity of dental bleaching treatments, ensuring a radiant smile for years to come [15].

All in all, patients are looking for a stunning smile that sparkles as a way to stand out from other people. In this manner, dental clinicians recommend patients should lessen the consumption of staining agents like coffee and tea, further, should not smoke or indulge in any supplementary habits that might produce tooth staining, particularly following tooth whitening, as some analyses have stated that bleaching products could alter the enamel surface by means of texture and morphology, consequently, making it more prone to dye absorption [20,21]. However, other studies suggest that the effectiveness of teeth whitening may not be directly influenced by diet [22,23]. To the best of the authors’ knowledge, there is no previous study describing a standardized diet that patients should follow after bleaching. Hence, the objective of this study is to assess through a systematic review and meta-analysis if a white diet during or after a bleaching treatment is needed. Accordingly, the null hypothesis of the current study was that the consumption of a free-staining diet after dental bleaching does not have an effect on the color change in enamel.

## 2. Materials and Methods

### 2.1. Data Sources

This systematic review and meta-analysis adheres to the guidelines outlined in the Preferred Reporting Items for Systematic Reviews and Meta-Analyses (PRISMA Statement) to ensure transparency and accuracy in reporting our research [24]. The study protocol was registered with PROSPERO under the code CRD42023437927. The following PICO framework was structured, focusing on the following aspects: population: bleached enamel; intervention: recommendation to adhere to a colorant-free diet during and after bleaching procedures; control: no restriction regarding the diet during or after the bleaching procedures; outcome: color difference, luminosity or whitening index; type of studies: in vitro studies and clinical trials. The core research question was as follows: is a white diet necessary during and/or after a bleaching treatment?

### 2.2. Search Strategy

The literature search was completed on 29 May 2023. Two independent reviewers, identified as R.B. and A.F.-L., were responsible for the search across multiple databases, including PubMed (MEDLINE), Cochrane Wiley, Web of Science, Scopus, EMBASE, and SciELO. The search strategy was devised according to the keywords detailed in Table 1, and all studies were managed through the Rayyan QCRI mobile app [25].

### 2.3. Eligibility Criteria

To determine which articles warranted full-text review, both R.B. and A.F.-L. independently assessed the titles and abstracts of identified articles. They applied specific criteria: the study had to be either an in vitro investigation or a clinical trial reporting on the color change in bleached enamel following a free-staining diet; inclusion of a control group where the diet was not restricted; evaluation of color difference following any bleaching protocol, regardless of the diet, luminosity, or whiteness index; provision of mean and standard deviation data for Delta E, luminosity, or whiteness index; and publication in English, Spanish, or Portuguese. Excluded were case reports, case series, pilot studies, expert opinions, conference abstracts, and reviews. Any disagreements during the study selection process were resolved through discussion and consensus with a third reviewer, CECS.

### 2.4. Data Extraction

Pertinent data from the selected manuscripts was extracted using Microsoft Office Excel 2016 software and compiled into a standardized form. Two reviewers, J.C.H.-C. and LH, both proficient in this software, independently conducted the data analysis. The extracted information included the first author, year of publication, bleaching agent applied, bleaching protocol used, staining agents tested, staining protocol used, and color measurement device.

### 2.5. Quality Assessment

The selected articles underwent a risk of bias assessment, categorized in accordance with appropriate tools to perform it: the Cochrane RoB2 tool for randomized clinical trials (Cochrane RoB2 tool) [26], and the RoBDEMAT tool for in vitro studies [27]. Two reviewers, C.E.C.-S. and R.B., conducted independent assessments of the articles.

### 2.6. Statistical Analysis

The meta-analysis was conducted using Review Manager software (version 5.3.5; the Cochrane Co., Copenhagen, Denmark). A random-effects model was employed for the analyses, and pooled-effect estimates were derived by comparing the standardized mean difference of the color parameters of bleached enamel when patients adhered to a restricted diet versus when they did not. Separate analyses were conducted for in-office or at-home bleaching procedures, with subgroups formed based on the staining agent tested. Statistical significance was set at a *p*-value < 0.05. Heterogeneity was assessed using the Cochran Q test and the inconsistency I2 test.

## 3. Results

A comprehensive search across multiple databases yielded a total of 3639 documents. After meticulously removing duplicate entries, 2865 unique articles were left for initial assessment based on their titles and abstracts. Subsequently, a thorough screening of titles and abstracts led to the identification of 39 studies that warranted a full-text examination.

Upon closer examination, 22 of these studies were excluded for the following reasons: in 8 studies, no pigments were used during or after the bleaching process [28,29,30,31,32,33,34,35], 5 studies lacked a control group [14,16,36,37,38], in 4 studies, the color difference, luminosity or whiteness index was not calculated [4,39,40,41], in 4 studies, the full text could not be retrieved [42,43,44,45], and 1 study was a review [23]. This process left 17 documents eligible for qualitative analysis: 5 clinical trials [46,47,48,49,50] and 12 in vitro studies [21,51,52,53,54,55,56,57,58,59,60,61].

**Figure 1:**
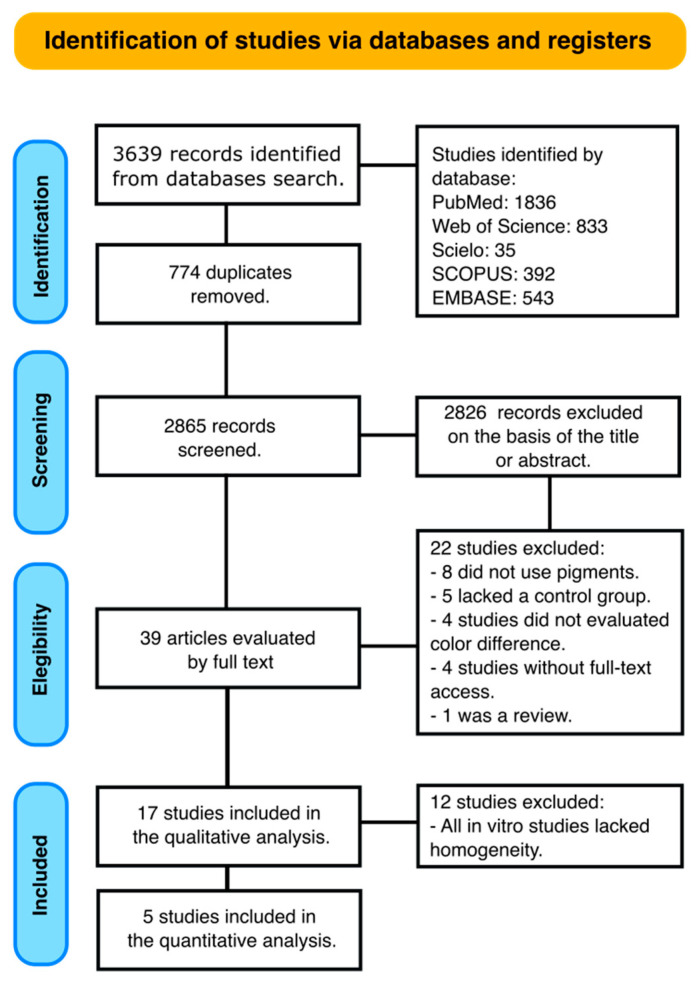
Study selection process according to the PRISMA statement guidelines.

After the analysis of the in vitro studies, it was decided not to perform a meta-analysis due to lack of homogeneity regarding the bleaching protocol, the staining protocol and the follow-up. On the other hand, data from the clinical trials were included in the quantitative analysis. The study selection process followed the guidelines outlined in the PRISMA statement and is visually represented in Figure 1.

The characteristics of the studies included in the review are presented in Table 2 and Table 3. For in vitro studies, both at-home and in-office products were tested, including CP- and hydrogen peroxide (HP)-containing products. Among the staining agents, tea, red wine, coffee, grape juice, cola, chocolate milk, and soya sauce were tested.

Most of the studies focused only on the evaluation of the color difference, and in some cases, individual data from the L, a, and b values were presented. Only one study evaluated the whiteness index. On the other hand, some studies included the evaluation of the roughness, surface elemental analysis, microhardness, and mineral loss.

Regarding the clinical trials, four were catalogued as randomized clinical trials, while one lacked the randomization process. The numbers of participants ranged from 40 to 80, with a maximum follow-up of 1 month after the bleaching procedures. Both in-office and at-home bleaching procedures were tested, using products based on carbamide peroxide and hydrogen peroxide. Regarding the staining agents, coffee, tea, red wine, and cola-based drinks were tested. In these studies, the main outcome was the color difference, and one study reported the whiteness index. Tooth sensitivity was evaluated in all the clinical trials included in the review.

**Figure 2:**
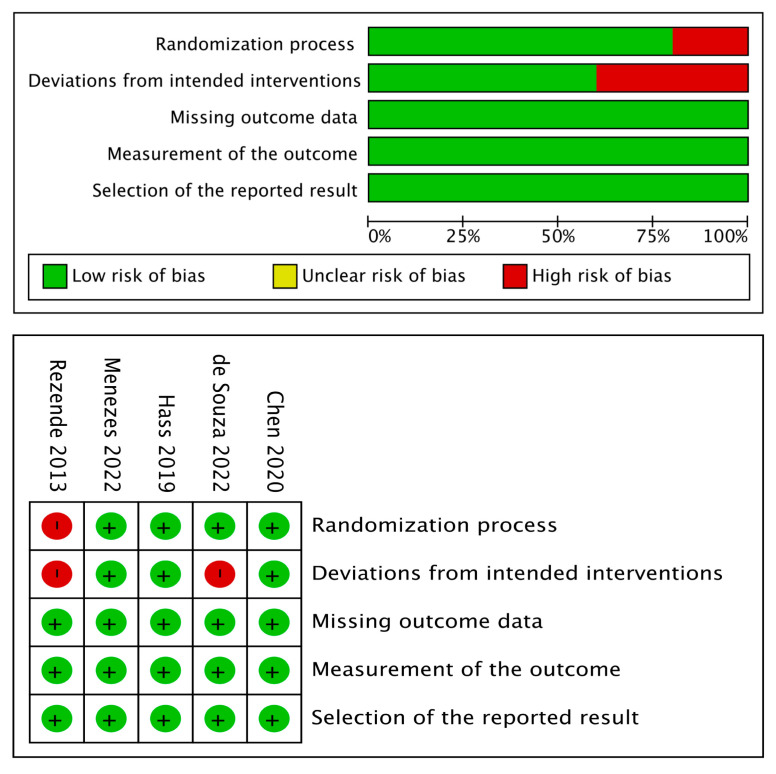
Risk of bias summary of clinical trials.

Table 4 presents the results of the risk of bias assessment for the in vitro studies. The majority of the studies exhibited shortcomings in sample size determination and blinding of the operator parameter. As for the clinical trials, the risk of bias determination results is depicted in Figure 2; in general, all studies were catalogued as low risk in the parameters evaluated, except for the domains of randomization and deviations from intended results.

**Figure 3:**
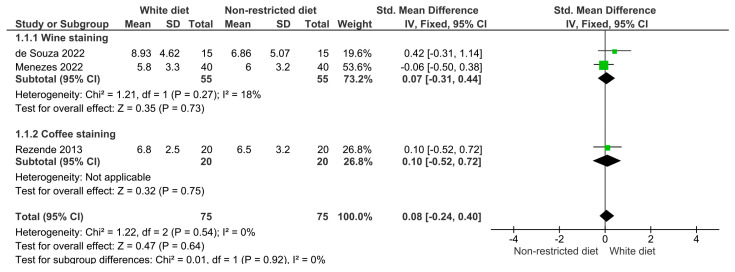
Forest plot showing the color difference in patients on a restricted diet versus patients who rinsed their teeth with wine or coffee during the first week of at-home whitening treatment.

**Figure 4:**
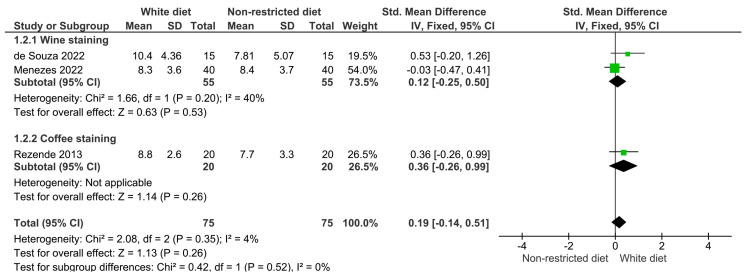
Forest plot showing the color difference in patients on a restricted diet versus patients who rinsed their teeth with wine or coffee during the second week of at-home whitening treatment.

**Figure 5:**
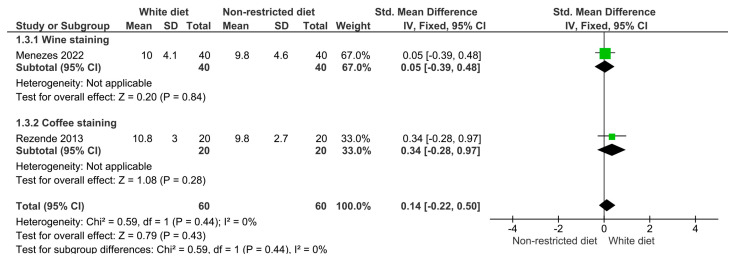
Forest plot showing the color difference in patients on a restricted diet versus patients who rinsed their teeth with wine or coffee during the third week of at-home whitening treatment.

**Figure 6:**
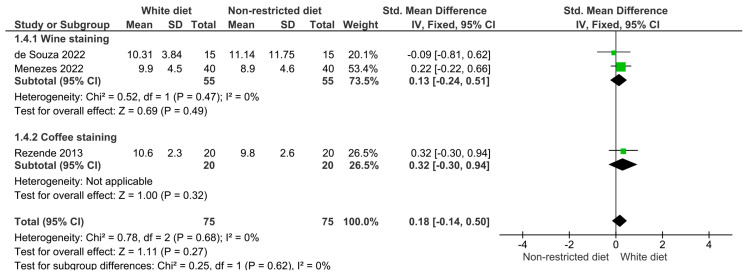
Forest plot showing the color difference in patients on a restricted diet versus patients who rinsed their teeth with wine or coffee one month after finishing bleaching treatment.

**Figure 7:**
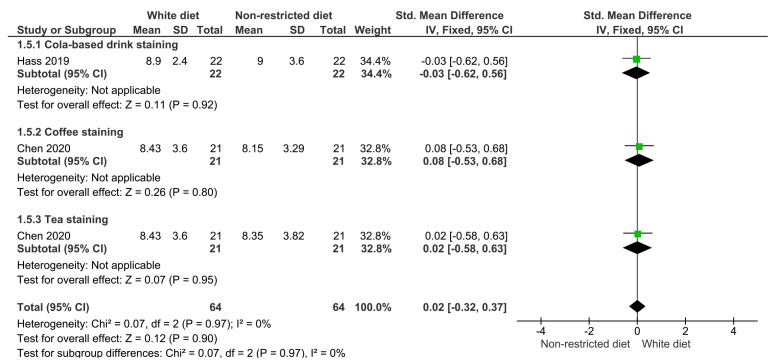
Forest plot showing the color difference in patients on a restricted diet versus patients who rinsed their teeth with cola-based soft drink, coffee, or tea one month after finishing bleaching treatment.

The results of the meta-analysis are illustrated in Figure 3, Figure 4, Figure 5, Figure 6 and Figure 7. Figure 3 shows the analysis of the color differences in the at-home bleaching procedures after the first week of treatment; according to this, the differences between the group with a restricted diet and the group where wine or coffee were administered to the experiment subjects were not statistically significant (*p* = 0.64). The same behavior was observed for the analyses after two and three weeks of treatment (Figure 4, *p* = 0.26; and Figure 5, *p* = 0.43). Figure 6 shows that differences in color difference one month after finishing the bleaching treatment were not statistically significant (*p* = 0.27).

Figure 7 displays the findings of the meta-analysis comparing the color difference one month after finishing an in-office treatment when the patients consumed cola-based soft drinks, coffee, or tea. According to the results, the diet restrictions did not significantly improve the bleaching outcomes (*p* = 0.90).

## 4. Discussion

This systematic review and meta-analysis aimed to assess the necessity of a white diet during and after a bleaching treatment. The whole findings for clinical and in vitro studies demonstrated that a white diet is not necessary during or after bleaching procedures. Given this focus, the null hypothesis tested in this study was accepted.

There remains ongoing debate among dentists not only regarding the dental bleaching technique itself but also regarding the post-operative care instructions provided to patients. One area of contention involves the restrictions imposed on the consumption of certain foods and beverages during and after bleaching, as well as the duration for which patients should avoid these items to ensure the long-term success of the treatment [58,62,63].

Plenty of studies have correlated the vulnerability to staining solution following bleaching with the variations produced by the whitening agents to the enamel structure [21,39,42,64,65,66,67]. These substances, often acidic in nature, have the potential to dissolve the mineral composition of the enamel, leading to the loss of calcium and phosphate and resulting in reduced crystal size and enlarged inter-crystalline gaps within the enamel [58]. During the dissolution process, the carbonate present in the enamel structure may also be lost, exposing the protein structure near the crystal matrix [68]. In this condition, the enamel becomes more susceptible to the infiltration of staining constituents [58]. Therefore, clinicians frequently advise patients to refrain from smoking and consuming tea, coffee, juice, or red wine during active bleaching procedures, with some companies recommending patients to adhere to a white diet during this period.

Based on the findings of this meta-analysis, the use of a free-staining diet during and after bleaching procedures is not deemed mandatory. Many individuals consume tea, coffee, cola, and red wine, which may contain staining agents, as part of their daily lives. While some authors have suggested that coffee could potentially negatively impact the bleaching process, others have found no clinical evidence of coffee staining following dental bleaching [14,16]. According to Attia et al., it is advisable to avoid contact with coloring substances during tooth bleaching, because although treatment stability is not compromised during the process, consuming coffee after tooth bleaching has been found to reduce its effectiveness [42]. Concurrently, a previous report established that the predisposition to staining was amplified when the enamel came into contact with coloring agents, including red wine, following the whitening process [41].

The results of previous studies showed no pigment action throughout the dental bleaching process [19,22,58]. In particular, when the bleached tooth enamel was in contact with coffee, this did not influence the bleaching of the specimens. As a result, the prescription for a white diet to make enamel less vulnerable to coloring becomes pointless [59]. Bleaching teeth with CP does not make the enamel more susceptible to discoloration. These findings are comparable to those discoveries of other articles that colored beverages had no negative effect on the ultimate color of the teeth [22,23]. Further, it is compatible with the findings of this meta-analysis. A previous report [69] recognized that drinkers of color-based drinks might necessitate a particular post-treatment maintenance plan.

Accordingly, a white diet is not the only factor that affects the maintenance of the bleaching treatment. Implementing maintenance by using professionally suggested home-care approaches is important. Indeed, some patients may opt to enhance and maintain their perfected tooth color after dental bleaching by returning once or twice a year for touch-up conservation protocols at the dental office. On the other hand, individuals who prefer home oral hygiene-based approaches may opt for powered toothbrushes, as this protocol has been shown to remove more stains and plaque compared to manual toothbrushes alone [70,71,72]. Other bleaching agents on the market include over-the-counter products such as bleaching toothpastes, strips, and mouthwashes [73]. These agents might be inadequate when compared to home-bleaching [74,75]. However, because of their stain removal capacity, they can be used as an alternative for color maintenance after whitening when monitored by a dental expert [76]. Moreover, fluoridated bleaching gels, nano-carbonate apatite, or fractional CO_2_ laser may support the post-bleaching maintenance effect by inducing the remineralization fluoride acquisition of the enamel, or preventing stain absorption [77,78,79].

It is noteworthy to mention that color changes following dental bleaching can be expressed by different tools, with the most used being the standard shade guide [80]. This tool might be subjective and is influenced by many factors such as the age of the observer, the experience, and the lighting conditions [81]. The color can be measured objectively by a colorimeter concurring to the Commission Internationale de l’Eclairage (CIE), expressing the color in a color space of 3 axes: L* (lightness ranging from 0: black to 100: reflecting diffuser), a* (+a: red and -a: green), and b* (+b: yellow and -b: blue).

While the primary focus of this review may not have been to explore the biochemical mechanisms behind why a colored diet does not affect bleaching outcomes, it is an important consideration for clinicians seeking a deeper understanding of the topic. Vital bleaching is known to result in acidic etching of the enamel, rendering it more susceptible to the infiltration of staining constituents. Additionally, peroxide, a common bleaching agent, has the capability to break down double bonds and modify the organic composition of both enamel and dentin, with dentin being responsible for the yellow color of teeth [67,82]. While acid etching may not be the primary mechanism of color change, it remains an important factor to consider [7].

Furthermore, recent studies have shed light on the role of peroxide in altering the organic structure of dental tissues, thereby influencing the color of teeth. Peroxide has been shown to penetrate the enamel and dentin, where it interacts with organic molecules, including proteins and pigments, leading to changes in their structure and composition [83]. This biochemical process, coupled with the acidic environment created during bleaching, can result in modifications to the surface morphology and chemical composition of dental tissues, ultimately affecting their susceptibility to staining [84].

While the exact mechanisms underlying the interaction between peroxide and dental tissues are still being elucidated, it is clear that peroxide plays a significant role in the bleaching process. Understanding these biochemical mechanisms can provide valuable insights into why dietary restrictions may not significantly impact bleaching outcomes. By considering the biochemical changes induced by bleaching agents, clinicians can better advise patients on post-bleaching care and dietary recommendations, ensuring optimal long-term results [85]. In conclusion, while this review may not have extensively covered the biochemical mechanisms behind tooth bleaching, it serves as a valuable resource for clinicians seeking guidance on advising patients after vital bleaching procedures. By incorporating a deeper understanding of the biochemical processes involved in bleaching, clinicians can enhance their ability to educate and counsel patients effectively, ultimately improving patient outcomes and satisfaction [7].

This systematic review highlights the multifaceted landscape of research in the field of dental whitening. The lack of homogeneity among in vitro studies underscores the need for standardized methodologies and consistent reporting practices. Furthermore, the lack of clinical trials with extended follow-up periods raises questions regarding the durability and long-term effects of various whitening interventions. Furthermore, the absence of studies specifically measuring the whiteness index of the teeth adds another layer of complexity to the existing body of literature. As the investigators navigate through these research gaps, it becomes imperative for future investigations to address these limitations, fostering a more comprehensive understanding of dental whitening practices and their implications. This will not only contribute to the scientific rigor of the field but also guide clinicians and researchers towards more informed and evidence-based approaches in the pursuit of achieving optimal dental aesthetics.

## 5. Conclusions

In conclusion, the evidence suggests that a restricted diet may not lead to improved clinical outcomes in dental bleaching. Despite the common assumption regarding the impact of dietary choices on dental aesthetics, this research highlights the need for a deeper understanding of the factors influencing the effectiveness of dental bleaching interventions.

## Figures and Tables

**Table 1:** Keywords used for the literature search in PubMed.

Search	Terms
#1	Dental bleaching OR at-home bleaching OR bleached teeth OR tooth bleaching OR tooth whitening OR bleaching OR vital bleaching OR bleached enamel
#2	Cola-based soft drink O carbonated beverages OR cola OR soda OR diet OR red wine OR coffee OR staining beverage OR dyes OR food coloring agents OR staining drinks OR pigmenting agents OR staining solutions OR drink OR tea OR staining beverages OR food colorant
#3	Bleaching effectiveness OR staining OR color alteration OR color change OR staining power OR clinical effect OR tooth color OR intrinsic color OR color stability OR tooth discoloration
#4	#1 and #2 and #3

**Table 2:** Characteristics of the in vitro studies included.

Study and Year	Bleaching Agent	Bleaching Protocol	Staining Agents	Staining Protocol	Color Measurement Device	Primary Outcome	Secondary Outcome	Main Results
Attin, 2003 [51]	10% carbamide peroxide—VivaStyle (Vivadent, Schaan, Liechtenstein)	8 h application over 8 days	Tea	Immersion for 10 min for 8 days	Spectrophotometer (Pikkio, Medical High Technologies, Verona, Italy)	Color difference		The immersion of the specimens in tea did not affect the bleaching.
Barbosa, 2023 [52]	10% carbamide peroxide—Pola- night 10% (SDI) 37.5% hydrogen peroxide—Polaoffice + (SDI)	2 h application over 30 days 3 applications of 8 min with a 7-day interval	Red wine Coffee	Immersion for 45 min for 30 days	Vita Easyshade^®^ Advance 4.0 (Vita Zahnfabrik H. RauterGmbH & Co, Bad Säckingen, Germany)	Color difference	Roughness Surface elemental analysis	Immersion in red wine or coffee did not affect the bleaching process.
Briso, 2016 [53]	10% carbamide peroxide—Whiteness Perfect (FGM Produtos Odontologicos Ltd., Santa Catarina, Brazil)	4 h application over 14 days	Coffee Grape juice	Immersion for 10 min for 14 days	Ultraviolet–visible reflection spec- trophotometer (UV-2450, Shimadzu Corporation, Kyoto, Japan)	Color difference		Bleaching outcome was not affected by the consumption of staining agents.
Camara, 2018 [54]	16% carbamide peroxide—Whiteness Perfect (FGM Dental Products, Joinville, Brazil)	4 h application over 21 days	Coffee	Immersion for 15 min for 21 days	Vita Easyshade™ Advance 4.0 spectrophotometer (VITA Zahnfabrik H. RauterGmbH & Co, BadSäckingen, Germany)	Color difference		Coffee did not affect the bleaching outcome.
Carlos, 2016 [55]	40% hydrogen peroxide—Opalescence Boost PF 40% (Ultradent South Jordan, UT, USA) 10% carbamide peroxide—Opalescence PF10% (Ultradent)	3 applications of 40 min with a 7-day interval 1 h application over 15 days	Cola Coffee	Immersion for 30 min for 15 days	Easyshade Advance (VITA, Bad Säckingen, Germany)	Color difference	Roughness Microhardness	The staining solution either cola or coffee negatively affected the results of bleaching.
Correia, 2017 [56]	22% carbamide peroxide—Whiteness Perfect (FGM Dental Products, Joinville, Brazil)	1 h application over 15 days	Coffee Cola Tea Red wine Chocolate milk Soya sauce	Immersion for 5 min twice a day for 15 days	Spectrophotometer (Minolta CR-321, Japan)	Color difference		Only soya sauce negatively affected the bleaching outcomes.
de Araújo, 2013 [21]	10% carbamide peroxide—Whiteness Perfect (FGM Produtos Odontologicos Ltd., Santa Catarina, Brazil)	6 h application over 21 days	Cola soft drink Melted chocolate Red wine	Immersion for 1 h for 21 days	Spectrophotometer (UV-2450; Shimadzu Corp.)	Color difference	Mineral loss Microhardness	Staining solutions did not affect the bleaching outcomes.
Karadas, 2014 [57]	10% carbamide peroxide—Opalescence 10%, (Ultradent Products, USA)	6 h application over 14 days	Red wine Coffee Cola Tea	Immersion for 15 min, 6 h, 1 week and 1 month	Spectrophotometer (Shadepilot, DeguDent GmbH, Hanau, Germany	Color difference		Staining agents decreased the bleaching effectiveness.
Mori, 2015 [58]	35% hydrogen peroxide gel—Lase Peroxide Sensy (DMC, São Carlos, Brazil)	2 applications of 15 min with a 3-day interval	Coffee	Immersion for 30 min for 7 or 14 days	Easyshade^®^ (Vita-Zahnfabrik, Bad Säckingen, Germany)	Whiteness index and closeness to white	Enamel remineralization	The whiteness index was not influenced by coffee.
Lins-Filho, 2019 [59]	35% hydrogen peroxide—Whiteness HP 35% (FGM Dental Products)	3 applications of 40 min with a 7-day interval	Coffee Wine	Immersion for 5 min for 7 days	Easyshade (Vita, Brea, California, USA)	Color difference		The staining agents did not affect the color change.
Rezende, 2019 [60]	16% carbamide peroxide—Whiteness Perfect (FGM, Joinville, Santa Catarina, Brazil)	3 h application over 21 days	Beet Carmine Caramel Red 40 dye	Immersion for 5 min, twice a day, for 21 days	Spectrophotometer (Vita Zahnfabrik, BadSäckingen, Germany)	Color difference		Exposure to staining agents did not affect the bleaching efficacy.
Russo, 2010 [61]	25% hydrogen peroxide—Zoom 2 (Discus Dental, Culver City, CA, USA)	3 applications of 20 min	Coffee	Immersion for 1 h, 12 times per day, for 6 days	VITA Easyshade (VITA Zahnfabrik, Bad Säckingen, Germany)	Color difference		The staining with coffee did not affect the bleaching outcome

**Table 3:** Characteristics of the clinical trials included.

Study and Year	Type of Clinical Trial	Registration	Number of Participants	Bleaching Agent	Bleaching Protocol	Staining Agents	Staining Protocol	Color Measurement Device	Primary Outcome	Secondary Outcome	Main Results
Chen, 2020 [46]	Randomized double-blinded clinical trial	Clinical Trials Registry #NCT03933527	61 participants	40% hydrogen peroxide -Opalescence BOOST PF 40%, (Ultradent, USA)	2 sessions of 2 applications of 20 min with a 7-day interval	Coffee Tea	Rinse for 30 s, 4 times daily for 28 days.	Easyshade (Vita ZahnFabrik)	Whiteness index Color difference	Tooth sensitivity	Coffee or tea did not interfere with the color change in bleaching treatment
De Souza, 2022 [47]	Randomized clinical trial	ReBEC # RBR-7sv2g8r	45 participants	16% carbamide peroxide—Magic White	4 h application over 14 days	Red wine	Rinse for 5 min 3 times dailyfor 14 days.	Vita Easyshade (Vita Zahnfabrik, Bad Säckingen, Germany)	Color difference	Postoperative sensitivity Satisfaction of patients	Colorant-rich diets did not influence the performance of the bleaching treatment.
Hass, 2019 [48]	Randomized single-blinded clinical trial	ReBEC # RBR-2nz5s2	44 participants	35% hydrogen peroxide—Whiteness HP Automixx 35 (FGM Dental Products)	2 sessions of 3 applications of 15 min with a 7-day interval15 min of HP application (2 sessions and 3 applications each)	Coca- Cola	Rinse for 30 s, 4 times daily for 30 days	Vita Easyshade (Vident, Brea, CA, USA).	Color difference	Tooth sensitivity	Cola-based soft drinks did not affect the bleaching outcome.
Menezes, 2022 [49]	Non-randomized clinical trial	ReBEC #RBR-3 × 9m5j	80 participants	10% carbamide peroxide—Whiteness Perfect (FGM Dental Products, Joinville, Brazil)	4 h application over 21 days	Red wine	Rinse for 30 s, 4 times daily for 21 days.	Vita Easyshade (Vita Zahnfabrik, Bad Säckingen, Germany)	Color difference	Tooth sensitivity Nitric oxide levels in saliva	Red wine does not influence the bleaching outcome
Rezende, 2013 [50]	Non-randomized clinical trial	Not mentioned	40 participants	16% carbamide peroxide—Whiteness Perfect, (FGM Dental Products, Joinville, Brazil)	3 h application over 21 days	Coffee	Rinse for 30 s, 4 times daily for 21 days.	Easyshade (Vita Zahnfabrik)	Color difference	Tooth sensitivity	Coffee consumption during dental bleaching did not influence the efficacy of bleaching

**Table 4:** Risk of bias analysis of in vitro studies.

Author	D1: Bias in Planning and Allocation			D2: Bias in Specimen Preparation		D3: Bias in Outcome Assessment		D4: Bias in Data Treatment and Reporting	
	Control Group	Randomization of Samples	Sample Size	Standardization of Samples and Material	Identical Experimental Conditions Across Groups	Adequate and Standardized Testing procedures and Outcomes	Blinding of the Test Operator	Statistical Analysis	Reporting Study Outcomes
Attin, 2003 [51]	√	Χ	Χ	√	√	√	Χ	Insufficient √	√
Barbosa, 2023 [52]	√	√	Χ	√	√	√	Χ	√	√
Briso, 2016 [53]	√	√	Χ	√	√	√	√	√	√
Camara, 2018 [54]	√	Χ	Χ	√	√	√	Χ	√	√
Carlos, 2016 [55]	√	√	√	√	√	√	√	Insufficient √	√
Correia, 2017 [56]	√	Χ	√	√	√	√	Χ	Insufficient √	√
de Araújo, 2013 [21]	√	Χ	Χ	√	√	√	Χ	Insufficient √	√
Karadas, 2014 [57]	√	√	Χ	√	√	√	Χ	Insufficient √	√
Mori, 2015 [58]	√	√	Χ	√	√	√	Χ	√	√
Lins-Filho, 2019 [59]	√	√	Χ	√	√	√	Χ	√	√
Rezende, 2019 [60]	√	√	Χ	√	√	√	Χ	Insufficient √	√
	√	√	Χ	√	√	√	Χ	√	√

Reported, √; not reported, Χ.

## Data Availability

Dataset available on request from the authors.
